# Supplementary material for: Sensitivity analysis for causality in observational studies for regulatory science
Source: J Clin Transl Sci. 2023 Dec 5;7(1):e267. doi: 10.1017/cts.2023.688 (PMC10877517; doi:10.1017/cts.2023.688)
Supplement: Díaz et al. supplementary material [file S205986612300688Xsup001.docx]

# Data structure and notation

The objective of this data analysis will be to estimate the causal effect of the treatment on the probability of developing a medical outcome 30 years after enrollment. The eligible medical records of the patients were reviewed, and the following information was extracted:

• Baseline variables
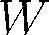
:

• Patient age at baseline

• Sex

• Date of first visit

• Xenodiagnosis at baseline (if conducted)

• Initial serology

• Physical examination and ECG tests within first 6 monthd from first visit

• Treatment
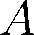
, with levels nifurtimox, benznidazole or control

• Indicator
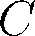
 of lost to follow-up. The criteria for lost to follow up are:

• Patients who expressed not to be followed up

• Patients who could not be reached to be followed up

• Patients who moved outside Santa Fe

• Patients who traveled to endemic areas

• Patients who became unable to attend medical examinations at the center

A patient’s outcome will be considered missing (
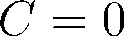
) if he/she was lost to follow-up during the study at any time point before
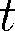
 years have passed, even if the medical outcome of interest has already occurred.

• Primary outcome
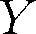
, an indicator of seroconversion having occurred before time
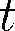
. This outcome is subject to missingness because some patients were lost to follow-up during the study.

• Secondary outcome
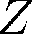
, an indicator of not having developed Chagas related ECG abnormalities before time
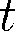
. This outcome is subject to missingness because some patients were lost to follow-up during the study.

The data of interest can be coded in terms of the following data structure:


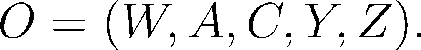
 (1)

To define the causal parameter of interest we use state-of-the-art methods for causal inference. Consider the following nonparametric structural equation model (NPSEM Pearl, 2000):


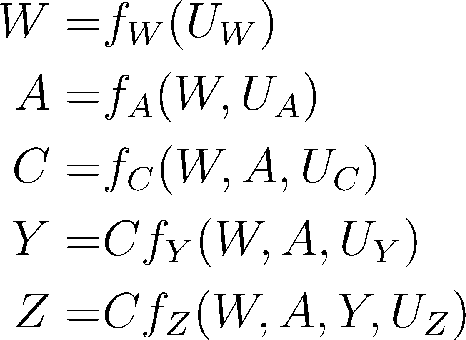
 (2)

which allows the definition of the counterfactual outcomes
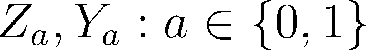
 corresponding to the outcomes in an intervened NPSEM in which the equations
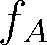
 and
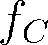
 are removed and
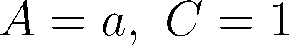
 are set deterministically. Thus,
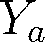
 represents the outcome of a subject if, possibly contrary to the fact, a subject would have received level
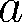
 of treatment and censoring was not present.

# Analysis for the primary endpoint of seroreversion

As previously mentioned, causal effects are defined in terms of the distribution of the counterfactual outcomes
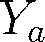
. For example,
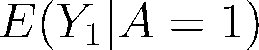
 denotes the cure rate among the treated if, contrary to the fact, we had treated and observed the outcome of all patients. The objective of this analysis is to estimate.


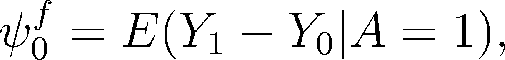


the causal effect of treatment among the treated. Under the randomization assumption that

 (3)

we have


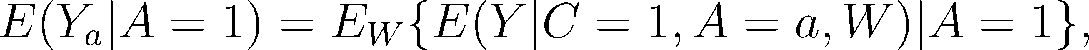


where the right-hand side only can be estimated from the observed data alone. However, since
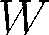
 may not contain all the confounding factors that determine missingness and treatment allocation, it is possible that the randomization assumption (3) does not hold. Therefore, we will perform a sensitivity analysis to measure the extent to which the efficacy of Nifurtimox can be established even under violations to this randomization assumption.

### Sensitivity analysis

This sensitivity analysis was first proposed by Díaz and van der Laan (2012). First, we will impute all the treated patients that were missing as treatment failures. As we will see, this provides a very conservative scenario for estimation of the causal effect of nifurtimox. The reason to use this conservative approach is that it yields a sensitivity parameter that is easy to interpret. Denote this imputed outcome by
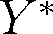
.

We will now address the problem of estimating
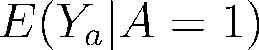
 for
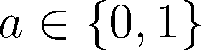
. Firstly, the counterfactual expectation
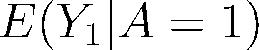
 can be conservatively approximated by
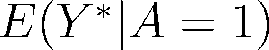
. That is


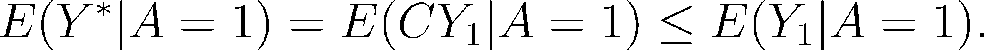
 (4)

As we will see, the use of this approximation will yield a sensitivity parameter with a more straightforward interpretation. Secondly, as previously mentioned, under violations to the randomization assumption (3)
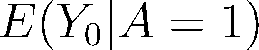
 is not equal to
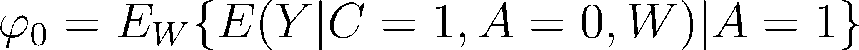
. However, we use
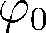
 as an approximation to
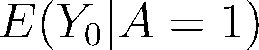
, and perform a sensitivity analysis on the amount of bias of this approximation. The value that best approximates
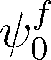
 is thus given by the estimand


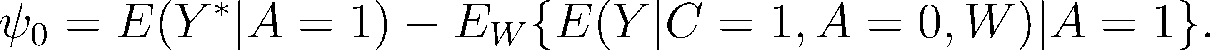
 (5)

Note that, using (4), the bias of this approximation of the desired causal effect
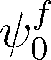
 is bounded above by


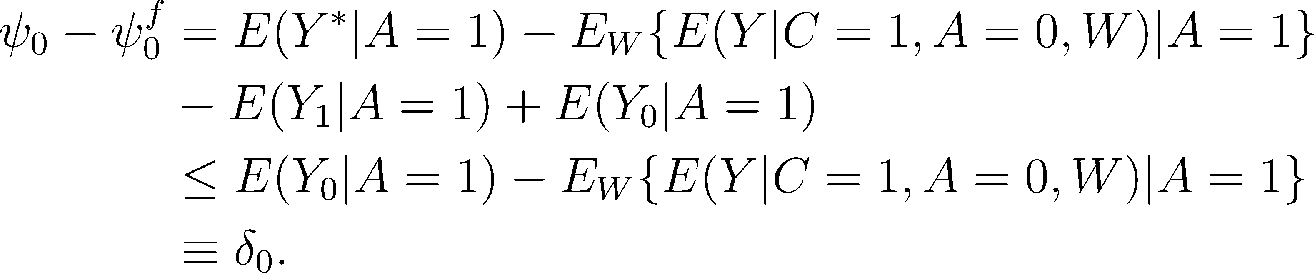


We use
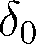
 as a sensitivity parameter to find the values of the bias
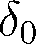
 for which the hypothesis of no treatment effect
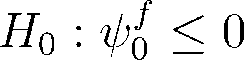
 would be rejected. An important feature of the sensitivity parameter
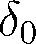
 is that under assumption (3)
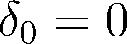
. Note also that


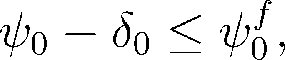


and therefore, for a given value of
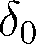
, the hypothesis of no efficacy of nifurtimox
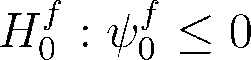
 implies the hypothesis
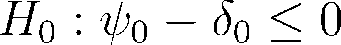
. Thus, rejecting
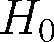
 implies rejecting
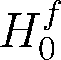
. For given
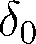
, if
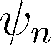
 is an asymptotically linear estimate of
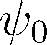
 with standard error
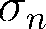
, we can use the statistic


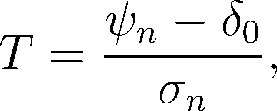


to define a valid test for
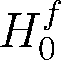
 as “*Reject
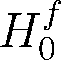
 if
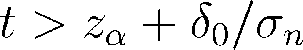
*”, where
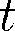
 is the observed value of
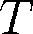
. Equivalently, given the value
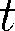
, we reject
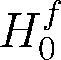
 when the true
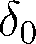
 satisfies


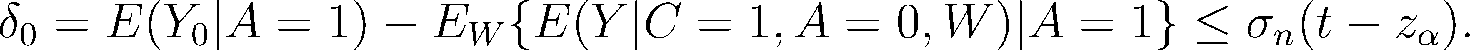


We can now plot the upper bound on
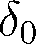
 that would lead to a rejection of
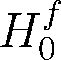
 as a function of the desired probability of type I error
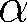
, and decide on the plausibility of each value of
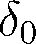
 based on subject-matter expert knowledge.

A targeted maximum likelihood estimator (van der Laan and Rubin, 2006; Rose and van der Laan, 2011) of
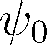
 and its variance estimation are discussed below.

## Targeted minimum loss-based estimation (TMLE) of the observed data parameters

The likelihood of the random variable
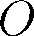
 in (1) can be factorized according to the previous time ordering as:


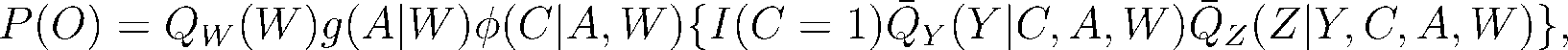
 (9)

where
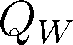
 is the marginal distribution of
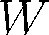
,
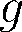
 is the distribution of
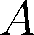
 given
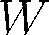
 (under randomization
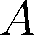
 would have been independent of
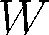
),
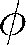
 denotes the missingness mechanism given
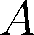
 and
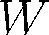
 (under complete MAR
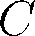
 would have been independent of
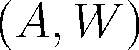
),
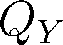
 is the seroconversion outcome distribution conditional on
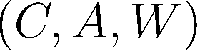
, and
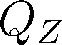
 is the Chagas related ECG abnormalities outcome distribution conditional on
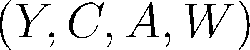
. We will denote the expectation of
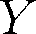
 conditional on
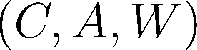
 by
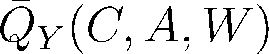
, the expectation of
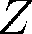
 conditional on
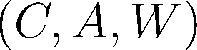
 by
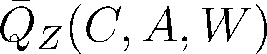
, and the expectation of
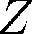
 conditional on
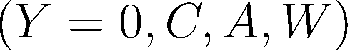
 by
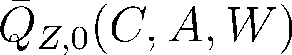
. Estimators of all the relevant quantities in the likelihood (9) will be performed through Super Learner (van der Laan et al., 2007), a machine learning algorithm that will be described in 6. The TML estimator that we present is discussed in more detail by van der Laan (2010) and Hubbard et al. (2011).

### TMLE of parameter (5) for the analysis of seroconversion

Recall the definition of estimand of interest:


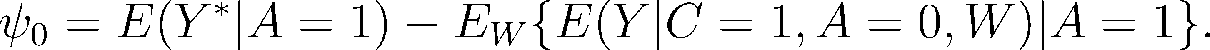


The expectation
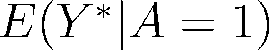
 will be estimated with its empirical counterpart, and a TMLE will be used to estimate
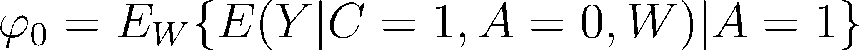
.

Implementation of the TMLE will be performed following the procedure described in chapter 8 of Rose and van der Laan (2011) as follows.

1. Find initial estimators
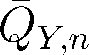
,
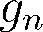
, and
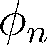
 of
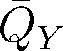
, , and .

2. Denote the number of treated patients. For each subject compute

where .

3. Estimate and in the univariate logistic regression models

4. Compute

5. Update and

6. Repeat 2-5 until convergence of .

The TMLE of is defined as the last value of this iterating process. In a slight abuse of notation, we will denote this converging value by . Assuming that the outcome, treatment, and censoring mechanisms are estimated consistently, under additional conditions explained in Appendix 18 of Rose and van der Laan (2011), is an asymptotically linear estimator with known influence curve :

where

Thus, the corresponding influence curve of the estimator of equals

and the variance of can be estimated as

where is the plug-in estimate of .

## Super learning

As explained in the previous subsection, the TMLE requires initial estimators of (treatment mechanism), (censoring mechanism), and (outcome mechanism). We will use an a priori-specified super learner for each one of these. Super learner is an ensemble learner that finds an optimal convex combination of a list of user-supplied estimators, based on cross-validation using the appropriate loss function (i.e, log-likelihood). It is optimal in the sense that it performs asymptotically as well as an oracle selector based on knowledge of the true distribution of the data. The finite sample size as well asymptotic properties of the super learner have been studied by van der Laan et al. (2007); van der Vaart et al. (2006), among others. The algorithms (Fixed before obtaining the data, immutable from then on) will be given by:

• Logistic regression with main terms

• Boosted logistic regression

• L1 regularized logistic regression

• Bayesian logistic regression with non-informative priors

• Generalized additive logistic regression models using smoothing splines with various degrees of freedom

• Boosted generalized additive logistic regression

• Sample mean

We will briefly describe the Super Learner algorithm; a complete description can be found in van der Laan et al. (2007). Consider the usual setting in which we observe identically distributed copies of the random variable . Super learner deals with estimation of parameters defined as the minimizer of the expectation of a loss function , the so-called risk of , over some parameter space . This is . Binary regression problems such as the case of the parameters , , , , and defined in the previous sections can be defined in these terms by using the logistic log-likelihood loss function.

An algorithm estimator of can be seen as a mapping that takes the empirical distribution and maps it into an estimate. is then the estimator based on the entire sample, and its conditional risk is given by

This conditional risk of an estimator depends on , and is therefore an unknown quantity that needs to be estimated. A first option is to use a plug-in estimator in which is used instead of . If the space is very large, this plug-in estimator of the risk will favor estimators that over-fit the data. Instead, super learner provides an algorithm that uses a v-fold cross validated risk estimate to choose the best estimator of .

Let index a random sample split into a validation sample and a training sample . Here we note that the union of the validation samples equals the total sample: , and the validations samples are disjoint: for . Let be the empirical distribution of the training sample , and let the empirical distribution of validation sample . The cross validated estimator of the risk is given by the following expression, in which the parameter is estimated on a training set and the risk is estimated in the corresponding validation set:

(10)

Assume that we have a list of candidate estimators . The discrete super learner is defined as the estimator in this list for which the cross validated risk in (10) is the smallest. Consider now a library of candidate estimators given by all possible convex linear combinations of the candidates . It can be shown (van der Laan et al., 2007) that the candidate in this library with the smallest cross validated risk is be given by

where

(11)

subject to and for all . Here denotes the size of the validation sample .

References

Iván Díaz and Mark J. van der Laan. Sensitivity analysis for causal inference under unmeasured confounding and measurement error problems. *U.C. Berkeley Division of Biostatistics Working Paper Series*, 2012. URL <http://biostats.bepress.com/ucbbiostat/paper303>.

Diana Fabbro, Mirtha L. Streiger, Enrique D. Arias María L. Bizai, Mónica del Barco, and Norberto A. Amicone. Trypanocide treatment among adults with chronic Chagas disease living in santa fe city (argentina), over a mean follow-up of 21 years: parasitological, serological and clinical evolution. *Revista da Sociedade Brasileira de Medicina Tropica*, 40 (1): 1–10, 2007.

Alan E. Hubbard, NicholasP. Jewell, and MarkJ. Laan. Direct effects and effect among the treated. In *Targeted Learning*, Springer Series in Statistics, pages 133–143. Springer New York, 2011. ISBN 978-1-4419-9781-4.

J. Pearl. *Causality: Models, Reasoning, and Inference*. Cambridge University Press, Cambridge, 2000.

S. Rose and M.J. van der Laan. *Targeted Learning: Causal Inference for Observational and Experimental Data*. Springer, New York, 2011.

Mark J. van der Laan. Estimation of causal effects of community based interventions. *U.C. Berkeley Division of Biostatistics Working Paper Series*, 2010. URL http://works.bepress.com/mark_van_der_laan/228.

M.J. van der Laan and D. Rubin. Targeted maximum likelihood learning. *The International Journal of Biostatistics*, 2 (1), 2006.

M.J. van der Laan, E. Polley, and A. Hubbard. Super learner. *Statistical Applications in Genetics and Molecular Biology*, 6 (25), 2007. ISSN 1.

A.W. van der Vaart, S. Dudoit, and M.J. van der Laan. Oracle inequalities for multi-fold cross-validation. *Statistics and Decisions*, 24 (3): 351–371, 2006.
